# Catheter-associated urinary tract infections in the intensive care unit during and after the COVID- 19 pandemic

**DOI:** 10.1186/s12879-025-10996-2

**Published:** 2025-04-24

**Authors:** Jakub Sleziak, Marta Błażejewska, Wiesława Duszyńska

**Affiliations:** 1 Department and Clinic of Anaesthesiology and Intensive Therapy, Wroclaw Medical University, L. Pasteura Street 1, Wroclaw, 50 - 367 Poland; 2 The Students Scientific Association by Department and Clinic of Anaesthesiology and Intensive Therapy, Wroclaw Medical University, Pasteura Street 1, 50-367 Wroclaw, Poland

**Keywords:** Urinary tract infections, CAUTI, ICU, Mortality, LOS, UTI prevention, HAI, Microbiological resistance

## Abstract

**Background:**

Urinary tract infections (UTIs) acquired in hospitals, particularly among patients in intensive care units (ICUs), are prevalent and represent a significant clinical issue as they are associated with increased patient morbidity, prolonged hospital stays, elevated healthcare costs, and antimicrobial resistance development.

**Methods:**

This study was conducted in the ICU of the University Hospital in Wrocław, Poland, from January 1, 2020, to June 30, 2024, and included 2,751 patients. The aim was to evaluate the incidence, epidemiological, and microbiological characteristics, mortality rates, and prevention strategies of UTIs during and after COVID-19 pandemic.

**Results:**

Catheter-Associated Urinary Tract Infection (CAUTI) (48 hours after admission) was recorded in 243/2751 (8.83%) patients, whereas UTI at admission was found in 63/2751 (2.3%). The mean CAUTI incidence rate (per 1,000 patient-days) was 6.99, 95% CI (6.13-7.85), whereas the mean CAUTI incidence density (per 1,000 urinary catheter days) was 7.04, 95% CI (6.18-7.91). CAUTI was significantly more frequent in females (12.32%) than in males (6.85%), *p* = 0.0000008, and in internal-medical patients (14.07%) compared to surgical patients (6.45%), *p* < 0.000001. The mean CAUTI density during the pandemic equaled 8.03, 95% CI (6.46-9.60) and was greater than in the post-pandemic period 6.25, 95% CI (5.34-7.17), *p* = 0.051. No statistically significant difference was observed in CAUTI incidence between COVID-positive 27/264 (10.23%) and COVID-negative 83/904 (9.18%) patients, *p* = 0.942. The most frequently identified pathogen in CAUTI was *Klebsiella pneumoniae* ESBL/MBL, whereas in UTI at admission, it was *Escherichia coli*. The percentage of alert pathogens among CAUTI etiological factors was significantly higher during the pandemic 72/116 (62.07%), compared to the post-pandemic period 62/143 (43.36%), *p* = 0.002. There was no statistically significant difference in mortality between CAUTI patients 56/207 (27.05%) and patients without CAUTI 810/2,544 (31.84%), *p* = 0.119.

**Conclusion:**

Although there were no statistically significant differences observed in CAUTI incidence between COVID-positive and COVID-negative patients, CAUTI remains a significant challenge in the ICU setting, with particularly elevated risks among female and internal-medical patients. Consistent monitoring of CAUTI, implementation, evaluation of preventive measures, and ongoing assessment are essential for improving clinical outcomes.

## Introduction

Healthcare-associated infections (HAIs) represent a significant challenge in medical settings, with urinary tract infections (UTIs) ranking as one of the most prevalent subtypes, almost completely stemming from urinary tract instrumentation [1]. Catheter-associated urinary tract infection (CAUTI) is one of the leading manifestations of HAIs globally, comprising up to 35% of all such infections [2]. This high prevalence can be partly explained by the fact that 15–25% of hospitalized patients undergo urethral catheterization during their hospital stay [3]. Studies show that the daily risk of bacteriuria ranges from 3–10%, approaching 100% after 30 days of catheter use [4].

Intensive care units (ICUs) exhibit the highest prevalence of HAIs [5]. Around 4% of ICU patients with stays exceeding two days develop UTIs, with 97% of these being CAUTIs [6]. That population is particularly prone to this infection due to prolonged catheterization, immunosuppression, and frequent use of invasive devices. Assessing the evolving patterns of HAIs and antibiotic resistance over different periods and regions is crucial for comprehending this significant global health threat’s progression and enabling evidence-based decisions about intervention strategies. This necessity is further underscored by the fact that a substantial proportion of CAUTIS are considered preventable [7].

The emergence of the COVID- 19 pandemic has significantly impacted this landscape potentially compromising standard HAI prevention practices. During the pandemic’s initial phase, healthcare workers reported increased workload, staff deficits, and the inclusion of nontrained personnel in both ICUs and the infection control sector [8]. Moreover, the vast majority of infection preventionists’ duties were COVID- 19-related at the pandemic’s outset [8]. During the pandemic, multiple countries reported increases in ICU admissions and ICU bed occupancy rates [9], along with increased HAIs rates in patients with SARS-CoV- 2 infection [10, 11]. Due to the increased use of medical devices, including vascular catheters, endotracheal tubes, and urinary catheters, as well as the implementation of prolonged mechanical ventilation, extracorporeal membrane oxygenation, the administration of immunosuppressive medications such as tocilizumab, and other patient-dependent factors, COVID- 19-infected patients faced a greater risk of secondary bacterial and fungal HAIs including CAUTI. These infections were simultaneously identified as a preventable mortality risk factor in this group of patients [12, 13]. The elevated incidences of HAIs in ICUs during the pandemic were accompanied by a notable rise in the number of microorganisms showing resistance to various antimicrobials [14].

Given the significant burden of CAUTIs in ICUs and the exacerbating effects of the COVID- 19 pandemic on HAIs, it is justified and reasonable to conduct a comprehensive analysis of CAUTI incidence, etiology, mortality, patient characteristics, length of stay (LOS), and antibiotic resistance patterns. Understanding how these parameters evolved during and after the pandemic is crucial for infection prevention strategy optimization. While several studies have documented changes in HAIs during the pandemic, comprehensive analyses comparing CAUTI epidemiology, microbiology, and outcomes between pandemic and post-pandemic periods remain limited, particularly in Central European settings. This study aimed to fill a critical knowledge gap by comprehensively analyzing these parameters during the pandemic period (2020 - 2021) and the subsequent period (2022- 2024 June) in a single-center ICU setting. Understanding the dynamics of CAUTIs in these distinct time frames may provide valuable insights into the impact of the pandemic on infection rates and inform the development of targeted interventions to enhance patient outcomes and strengthen infection control practices in the ICU environment. These findings may have important implications for infection prevention protocols and resource allocation in similar healthcare settings.

## Materials and methods

### Design and data collection

Between January 1, 2020, and June 30, 2024, a prospective observational study was conducted at the Department of Anesthesiology and Intensive Therapy, Medical University of Wroclaw, Poland. This study included all ICU admissions during the specified period, encompassing 2791 patients. Infections were tracked through regular infection monitoring, relying on monthly ICU infection reports from the Infection Monitoring and Treatment Laboratory, hospital databases, and annual microbiological reports. Necessary information, such as the number of patients treated and the use of medical devices, was recorded daily on an infection surveillance card, which allowed to count both the patient days (Pt-D) and urinary catheter days (Uct-D) as well as the intensity of urinary catheter utilization ratio (Uct-UR). The primary aim was to determine the incidence rate of CAUTIs among ICU patients. This involved evaluating the incidence of CAUTIs during the ICU stay as well as the assessment of UTIs present during admission to the department. Patients’ characteristics were described by gender and the patient’s most recent medical history, resulting in the ICU admission (medical or surgical). UTI monitoring was based on key indicators according to the European Centre for Disease Prevention and Control (ECDC) protocol [15]. For the complete picture of UTI manifestations at the department, UTIs present at admission to the unit were also monitored, recorded, and then analyzed. Such infections were associated with indwelling urinary catheterization > 48 h in only a minimal percentage of cases when patients were transferred from other hospital units. The majority of UTIs present at admission were non-catheter-associated, occurring in patients admitted from beyond the hospital setting, with symptoms of urosepsis. The study examined microbiological factors associated with infections and assessed adherence to the CAUTI prevention protocol elements according to the Centers for Disease Control and Prevention’s (CDC) National Healthcare Safety Network (NHSN) guidelines. This information was gathered biweekly by trained students from the Students Science Club from January 2020 to February 2024.

### Clinical diagnosis of UTI

UTIs in catheterized patients were documented on the hospital infection monitoring card and diagnosed according to the guidelines of the ECDC and NHSN [1, 15]. The study included all patients with urinary catheters hospitalized for more than 48 hours. For a CAUTI diagnosis, patients must simultaneously satisfy three essential criteria.

First, they must have had an indwelling urinary catheter in place for more than two consecutive days as an inpatient, with the catheter either still present during the calendar day when the infection is identified or removed the day prior to the infection date. When the device was placed before admission, the counting of Ct-D begins on the admission date to the ICU. This practice ensures consistency with the device denominator count [16].

Second, the patient must exhibit at least one of the following signs or symptoms without another recognizable cause: fever exceeding 38 °C, urinary urgency, increased frequency, dysuria, or pain/tenderness in the suprapubic region and/or costovertebral angle. It is important to note that symptoms related to urinary urgency, frequency, dysuria, and pain are only considered valid diagnostic criteria in conscious patients not receiving sedation. The symptoms of urinary urgency, frequency, and dysuria specifically could not be used as diagnostic criteria when an indwelling urinary catheter was still in place, as the catheter itself bypasses normal urination and may cause sensations that could mimic these symptoms. In our assessment, these criteria were only applied to patients after catheter removal when appropriate. Also, fever was not possible to be observed during CRRT.

Third, the microbiological diagnostic criteria mandate a positive urine culture yielding $\ge$10^5^ colony-forming units (CFU) per milliliter of urine, with no more than two species of microorganisms identified. Additionally, urinalysis findings supporting the diagnosis include the presence of leukocyte clumps and a microscopic examination revealing more than 15 leukocytes per high-power field.

These diagnostic elements must be temporally related and occur within the designated Infection Window Period in accordance with the NHSN HAI surveillance definitions [1].

### Methods for microbial identification

Microbiological diagnostics were performed in the Microbiological Laboratory of University Hospital in Wroclaw. Pathogen identification, susceptibility testing, and resistance mechanisms were conducted according to established European/Polish microbiological diagnostic standards and European Committee on Antimicrobial Susceptibility Testing recommendations [17]. The identification and classification of multidrug-resistant bacterial strains named “alert pathogens” followed the definitions proposed by Magiorakos et al [18]. Alert pathogens included: Gram-negative bacteria (Enterobacteriaceae classes, *Pseudomonas aeruginosa*, *Acinetobacter* spp.) exhibiting multidrug-resistant (MDR), extensively drug-resistant (XDR), and pandrug-resistant (PDR) phenotypes, extended-spectrum beta-lactamase (ESBL) producers, AmpC producers, carbapenemase producers including metallo-beta-lactamase (MBL), Verona integron-encoded metallo-beta-lactamase (VIM), New Delhi metallo-beta-lactamase (NDM), and oxacillinase- 48 (OXA- 48), as well as Gram-positive bacteria displaying glycopeptide-resistant enterococci (GRE), vancomycin-resistant enterococci (VRE), methicillin-resistant Staphylococcus aureus (MRSA) and vancomycin-intermediate and vancomycin-resistant Staphylococcus aureus (VISA/VRSA).

### Epidemiological indicators

The epidemiological indicators of CAUTI were counted as follows:

$$\begin{aligned} \text {CAUTI Incidence Density} = \left( \frac{\text {Number of CAUTI cases}}{\text {Number of Uct-D}}\right) \times 1000 \end{aligned}$$

$$\begin{aligned} \text {CAUTI Incidence Rate} = \left( \frac{\text {Number of CAUTI cases}}{\text {Number of Pt-D}}\right) \times 1000 \end{aligned}$$

$$\begin{aligned} \text {CAUTI Frequency} = \left( \frac{\text {Number of CAUTI cases}}{\text {100 patients admitted to the ICU at 1 year}}\right) \times 100 \end{aligned}$$

$$\begin{aligned} \text {Uct-UR} = \left( \frac{\text {Number of Uct-D}}{\text {Number of Pt-D}}\right) \times 100 \end{aligned}$$

The adherence to CAUTI preventive measures bundles outlined by CDC guidelines was calculated as a percent of observed realized bundle points divided by the total number of observations performed [19].

### Statistical analysis

Statistical analyses were carried out using Microsoft^®^ Excel (Version 16.89.1) data analysis ToolPack. Categorical data were presented as counts and percentages, while quantitative data were displayed as mean ± standard deviation (SD), median ± interquartile range (IQR), or 95% confidence interval (CI). Qualitative data were compared between groups using the chi-square test or Pearson’s chi-square test with Yates corrections, as appropriate. Qualitative data analysis was performed with either a two-tailed student’s t-test or ANOVA-single factor depending on the number of compared groups. For analyzing the correlation relationship, Pearson’s correlation coefficient was calculated, followed by determining the t-statistic and subsequent conversion to *p*-values using Student’s t-distribution with n- 2 degrees of freedom. A *p*-value of less than 0.05 was considered statistically significant.

### Ethical approval

All information related to patients, along with the microbiology test results, were collected as part of routine patient care and infection monitoring. Throughout data collection and manuscript preparation, patient data confidentiality was strictly maintained. According to the guidelines set by the Bioethics Committee of the Wroclaw Medical University, formal documentation of consent and written declarations from patients were not required. This study was approved under reference number KB- 576/2016.

## Results

Over the four-and-a-half-year observational period, the ICU admitted 2751 patients, of which 1753 (63.72%) were male and 998 (36.28%) were female. Among these, 1891 (68.74%) were surgical patients, and 860 (31.26%) were medical. The patient characteristics for each year were presented individually in Table 1.

**Table 1:** Characteristics of patients in each year

Category	2020	2021	2022	2023	2024
No. of patients	570 (100%)	598 (100%)	572 (100%)	643 (100%)	368 (100%)
Female	210 (36.8%)	226 (37.8%)	205 (35.8%)	233 (36.2%)	124 (33.7%)
Male	360 (63.2%)	372 (62.2%)	367 (64.2%)	410 (63.8%)	244 (66.3%)
Surgical patients	339 (59.5%)	342 (57.2%)	431 (75.3%)	503 (78.2%)	276 (75.0%)
Medical patients	231 (40.5%)	256 (42.8%)	141 (24.7%)	140 (21.8%)	92 (25.0%)
Pt-D	6517	7553	8704	8154	4484
Uct-D	6446	7490	8652	8093	4453
Uc-UR	98.91	99.17	99.40	99.25	99.31

(% of the total number of patients in each year). Number of patient days (Pt-D), urinary catheter days (Uct-D), and urinary catheter utilization ratio (Uc-UR) in particular years

Throughout 35412 Pt-D of hospitalization and 35134 Uct-D, 243 (8.83%) cases of CAUTI were recorded (in 207 patients). 50.6% of CAUTI occurred in women (123/243), 49.4% in men (120/243), 50.2% in surgical patients (122/243), and 49.8% in medical patients (121/243). During the pandemic years of 2020 and 2021, the overall number of CAUTI cases - 110 did not differ significantly statistically compared to 133 cases in the following post-pandemic period (2022 - 2024 VI); *p* = 0.32. The highest mean frequency of CAUTI/100 admitted patients was recorded in 2021 and equaled 11.03, CI95% (7.61 - 14.45), and the lowest - 7.17, CI95% (3.12 - 11.23) was recorded in 2024. Table 2 shows the sex structure of CAUTI patients in particular years as well as patients’ disease characteristics.

**Table 2:** The number of catheter-associated urinary tract infections (CAUTIs) cases recorded in the examined period, broken down by gender (female and male) and patient type (surgical and medical)

CAUTI	2020	2021	2022	2023	2024
TOTAL	47 (100%)	63 (100%)	59 (100%)	49 (100%)	25 (100%)
in women	29 (61.7%)	28 (44.4%)	29 (49.2%)	24 (49.0%)	13 (52.0%)
in men	18 (38.3%)	35 (55.6%)	30 (50.8%)	25 (51.0%)	12 (48.0%)
in surgical patients	22 (46.8%)	24 (38.1%)	36 (61.0%)	28 (57.1%)	12 (48.0%)
in medical patients	25 (53.2%)	39 (61.9%)	23 (39.0%)	21 (42.9%)	13 (52.0%)

The mean CAUTI frequency/100 admissions in the entire studied interval equaled 9.27 CI 95% (8.06–10.47). The mean CAUTI incidence rate/1000 Pt-D throughout the observational period was 6.99 CI 95% (6.13–7.85) whereas the mean CAUTI incidence density/1000 Uct-D was 7.04 CI 95% (6.18–7.91). The aforementioned epidemiological indicators for particular years were listed in Table 3. CAUTI was significantly more frequent in females 123/998 (12.32%) than in males 120/1753 (6.85%) *p* = 0.0000008 and in medical patients 121/860 (14.07%), than in surgical 122/1891 (6.45%), *p* < 0.000001.

**Table 3:** Mean values of epidemiological indicators of catheter-associated urinary tract infections (CAUTIs) in particular years of the investigated period (95% Confidence Interval)

Indicator	2020	2021	2022	2023	2024 (I-VI)
CAUTI frequency	8.50	11.03	10.73	7.86	9.27
	(5.8–11.21)	(7.61–14.45)	(8.14–13.32)	(5.64–10.07)	(3.12–11.23)
CAUTI rate	7.31	8.59	6.78	5.97	5.58
	(5.06–9.56)	(6.12–11.06)	(5.15–8.42)	(4.34–7.60)	(3.47–7.69)
CAUTI density	7.4	8.66	6.81	6.02	5.61
	(5.11–9.69)	(6.18–11.13)	(5.18–8.44)	(4.37–7.67)	(3.52–7.70)

**Fig. 1:**
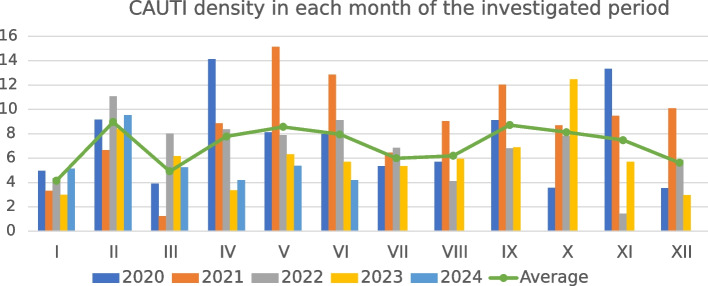
Catheter-associated urinary tract infections (CAUTI) incidence densities in separate months of particular years of investigated period along with the average CAUTI density value from all years for each month (green line)

CAUTI incidence densities in separate months of each of the years in the studied period are shown in Fig. 1.

### Comparison of the pandemic and post-pandemic period

A comparative analysis of the pandemic (2020 - 2021) and post-pandemic (2022 - 2024 I-VI) period was performed and is presented in Tables 4 and 5.

**Table 4:** Comparison of general hospitalization and catheter-associated urinary tract infection (CAUTI) parameters between pandemic (2020 - 2021) and post-pandemic (2022 - 2024 VI) periods, n, (95% Confidence Interval)

Parameter	Pandemic period	Post-pandemic period	*p* value
No. of hospitalized patients	1168	1583	0.154
Mean monthly Pt-D	586.25	711.4	0.00002
	(545.23–627.27)	(693.18–729.62)	
Mean monthly Uct-D	580.67	706.6	0.000002
	(539.8–621.51)	(688.10–725.10)	
Mean CAUTI frequency	9.77	8.87	0.462
	(7.69–11.84)	(7.38–10.36)	
Mean CAUTI rate	7.95	6.22	0.054
	(6.39–9.51)	(5.30–7.13)	
Mean CAUTI density	8.03	6.25	0.051
	(6.46–9.60)	(5.34–7.17)	

The analysis revealed that while the number of hospitalized patients remained stable across both periods, the post-pandemic period was characterized by higher Pt-D and Uct-D (pandemic: 14,070 Pt-D and 13,936 Uct-D vs. post-pandemic: 19,982 Pt-D and 21,198 Uct-D). Despite these increased care metrics, CAUTI frequency remained stable between the pandemic and post-pandemic periods. A trend toward improved CAUTI rates and density was observed in the post-pandemic period, though this improvement reached only marginal statistical significance.

**Table 5:** Catheter-associated urinary tract infection (CAUTI) incidence and mortality analysis in relation to patients’ COVID- 19 coinfection status during pandemic period (2020 - 2021), n/selected group (%)

Parameter	COVID-positive	COVID-negative	*p* value
CAUTI incidence^a^	27/264 (10.23%)	83/904 (9.18%)	0.942
Mortality in CAUTI patients^b^	12/29 (41.38%)	17/81 (20.99%)	0.035
Overall mortality^c^	108/264 (40.91%)	301/904 (33.3%)	0.016

^a^CAUTI incidence: number of CAUTI cases divided by the total number of patients in each COVID status group (COVID-positive: 27 CAUTI cases among 264 COVID-positive patients; COVID-negative: 83 CAUTI cases among 904 COVID-negative patients)

^b^Mortality in CAUTI patients: the number of deaths among patients with CAUTI divided by the total number of CAUTI cases in each COVID status group (COVID-positive: 12 deaths among 29 CAUTI cases; COVID-negative: 17 deaths among 81 CAUTI cases)

**c**Overall mortality: total number of deaths divided by the total number of patients in each COVID status group (COVID-positive: 108 deaths among 264 COVID-positive patients; COVID-negative: 301 deaths among 603 COVID-negative patients)

During the pandemic period, while CAUTI incidence was comparable between COVID-positive and COVID-negative patients, COVID-positive status was associated with significantly higher mortality among patients who developed CAUTI. This increased mortality in COVID- 19-positive patients with CAUTI appeared to be primarily driven by the overall higher mortality associated with COVID- 19 infection rather than the presence of CAUTI itself, as evidenced by similar mortality rates between COVID- 19-positive patients regardless of CAUTI status.

### Microbiological analysis

**Fig. 2:**
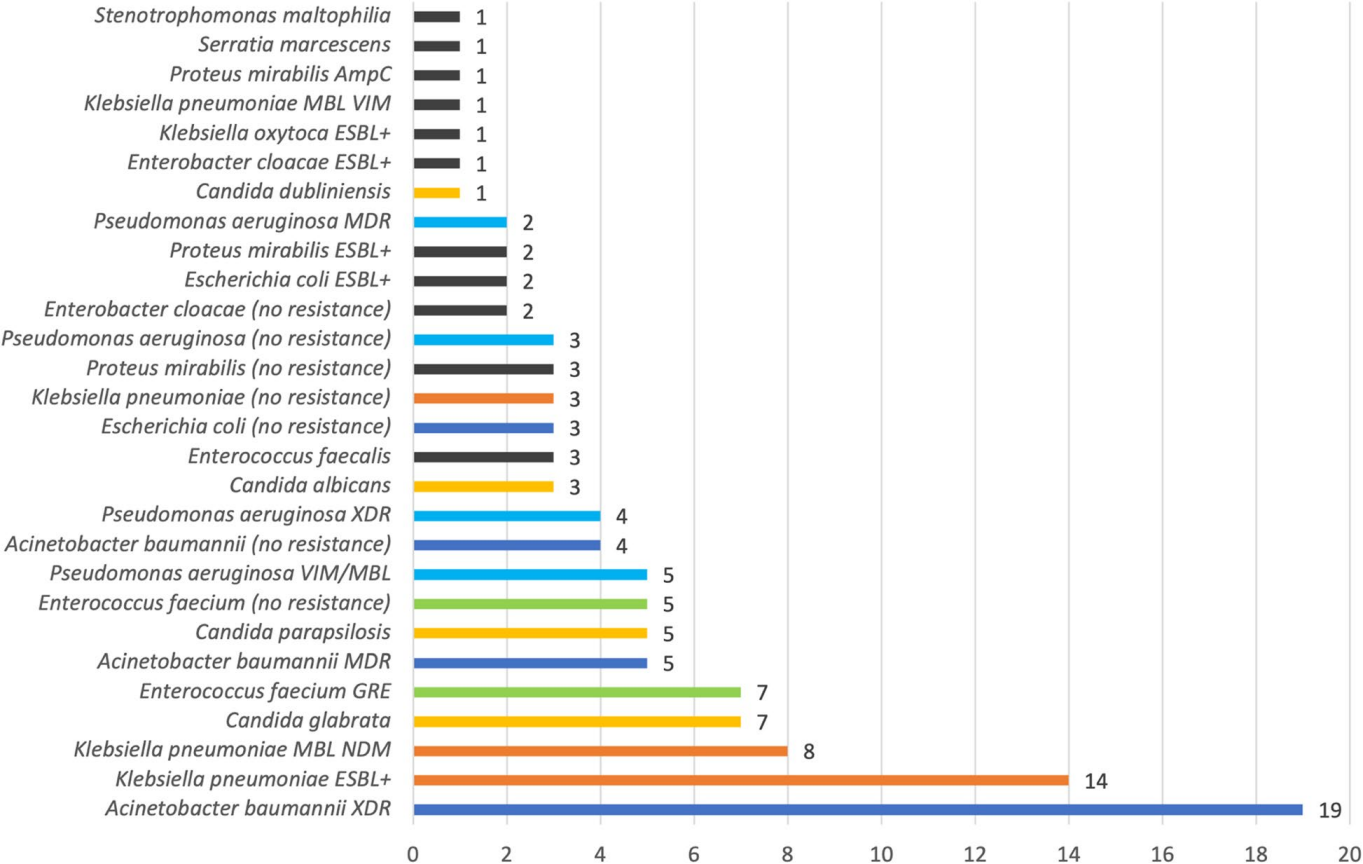
The total number of particular catheter-associated urinary tract infection (CAUTI) etiological factors isolated in microbiological analyses during the pandemic

**Fig. 3:**
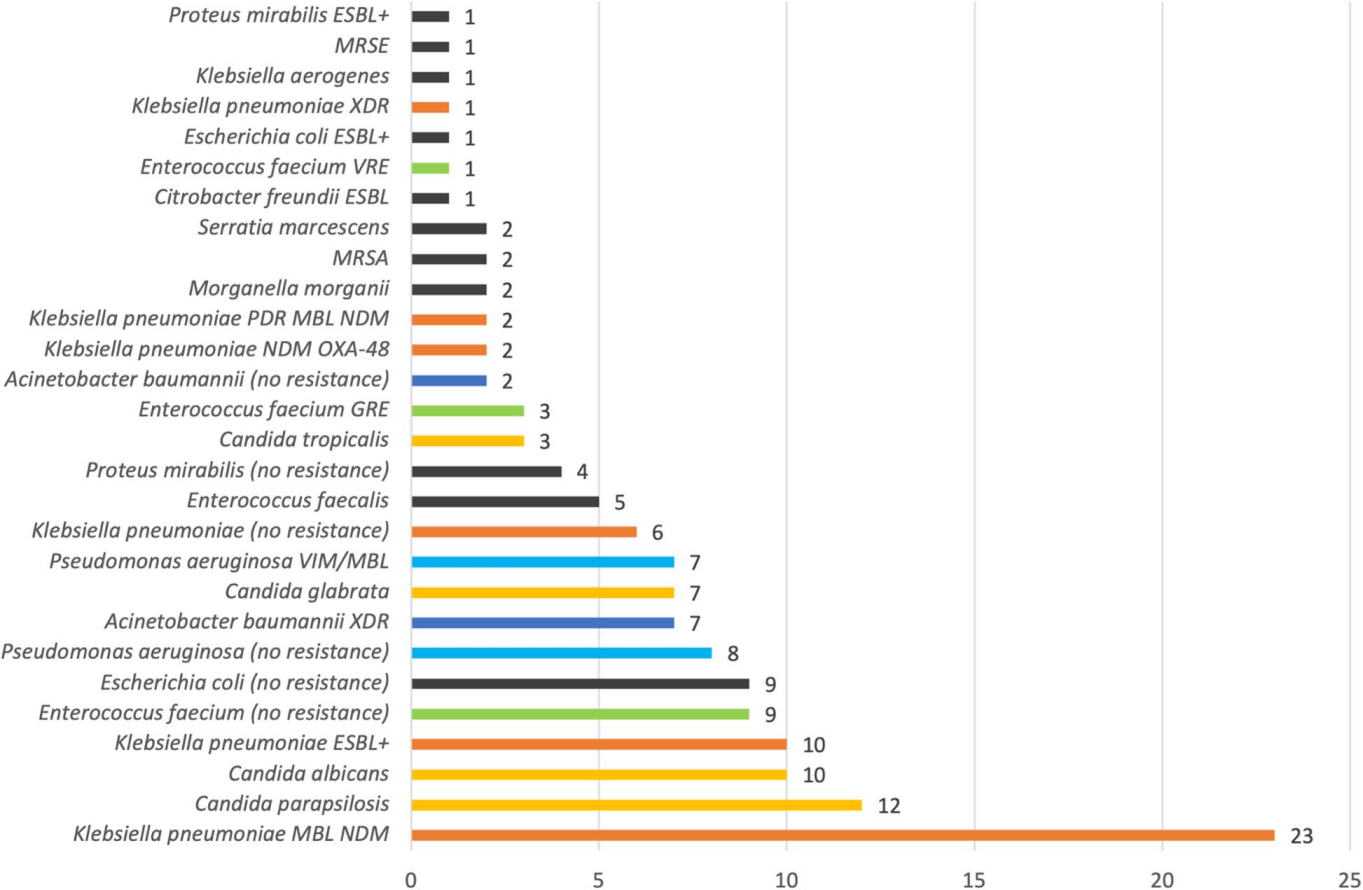
The total number of particular catheter-associated urinary tract infection (CAUTI) etiological factors isolated in microbiological analyses after the pandemic

Another key aspect of this study was the assessment of CAUTI etiological factors. The microbiological analysis comprised 259 findings classified as CAUTI causative pathogens. The most commonly isolated pathogenic organism throughout the total period was *Klebsiella pneumoniae* 70/259 (27.03%), followed by *Candida* spp. 48/259 (18.53%), *Acinetobacter baumannii* 37/259 (14.29%) and *Pseudomonas aeruginosa* 29/259 (11.2%). Figures 2 and 3 represent the most predominant etiological factors during and after the pandemic.

**Fig. 4:**
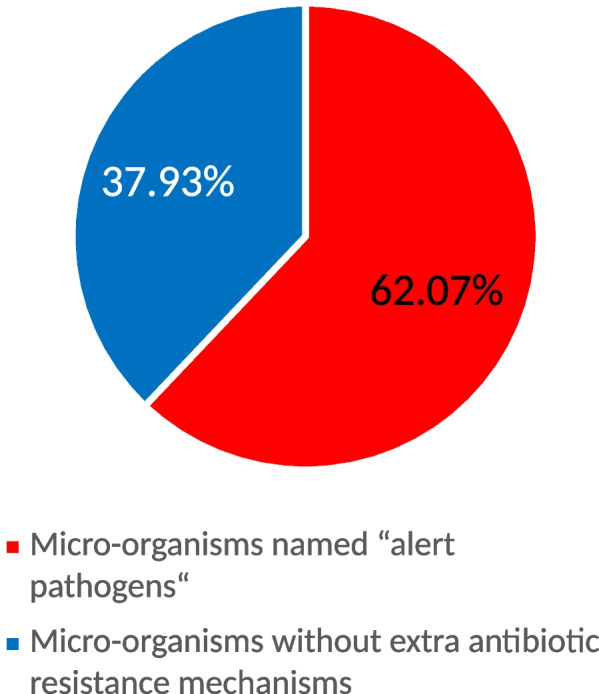
Percentage of alert pathogens in catheter-associated urinary tract infection (CAUTI) etiological factors during the pandemic

**Fig. 5:**
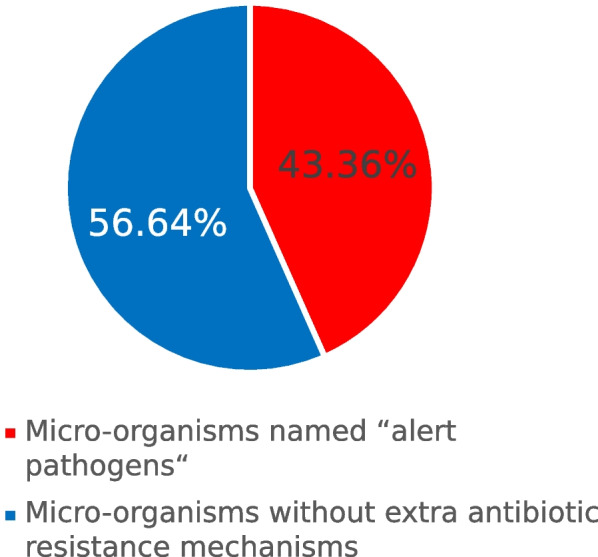
Percentage of alert pathogens in catheter-associated urinary tract infection (CAUTI) etiological factors after the pandemic

Of all etiological factors, the overall percentage of “alert pathogens” equaled 134/259 (51.74%). The ESBL resistance mechanism was found in 33/259 (12.74%) and MBL in 44/259 (16.99%). This percentage of alert pathogens was significantly higher in pandemic times 72/116 (62.07%), compared to post-pandemic 62/143 (43.36%), *p* = 0.002. The CAUTI of *Candida* spp. etiology was found with equal frequency during and after the pandemic, *p* = 0.08. Antibiotic resistance patterns in both periods are presented in Figs. 4 and 5.

### Mortality analysis

There was no statistically significant difference in mortality of CAUTI 56/207 (27.05%) vs patients without CAUTI 810/2544 (31.84%), *p* = 0.119. Further CAUTI mortality assessment revealed no significant difference among either males 32/103 (31.07%) and females 24/104 (23.08%), *p* = 0.196, or surgical 24/109 (22.02%) and medical 32/98 (32.65%), *p* = 0.075. No statistical significance was found in the mortality of patients with CAUTI caused by alert pathogens 24/90 (21.05%) compared to CAUTI caused by bacteria of standard antibiotic susceptibility 16/59 (27.12%) *p* = 0.44.

### Length of stay analysis

**Fig. 6:**
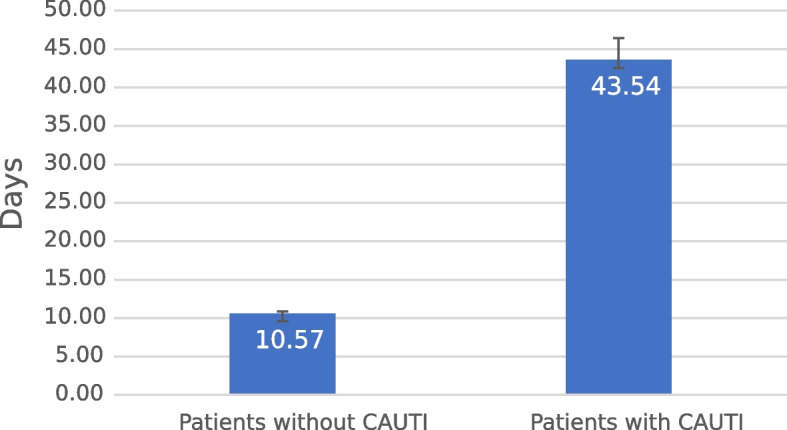
The mean length of hospitalisation at ICU in patients with and without catheter-associated urinary tract infection (CAUTI) presented in days depicted along with the standard error

The next point of interest in this study was the evaluation of the duration of hospitalisation. The median duration of hospitalization was 7 days (IQR: 4–15). The mean LOS at the ICU for patients in the total period equaled 13.02 days CI 95% (12.32–13.71), and no statistically significant differences were observed between particular years, *p* = 0.09. The LOS of patients afflicted with CAUTI (mean: 43.54 CI95% [37.92–49.16]; median: 36 [IQR 19–52]) was significantly longer than the LOS of patients without CAUTI (mean: 10.57 CI95% [10.08–11.07]; median 6 [IQR 3–13]), *p* < 0.0000001. This relationship is pictured in Fig. 6. The statistical analysis of LOS revealed that CAUTI caused by alert pathogens occurred on average around 9 days later, *p* = 0.036. The median day of the first CAUTI diagnosis was 20 IQR (12–29).

### Analysis of urinary tract infections present at admission

**Fig. 7:**
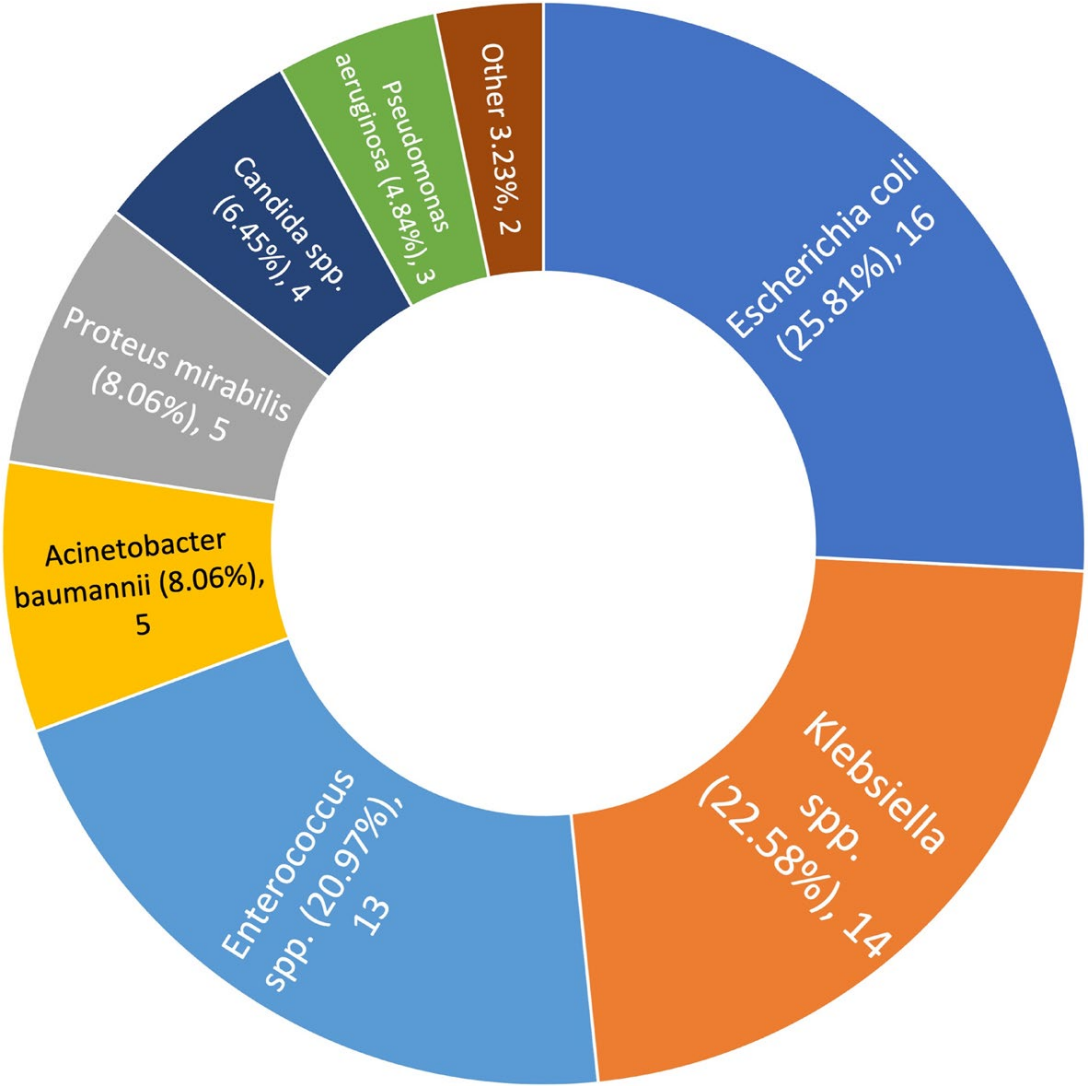
The pathogesns identified as etiological factors of urinary tract infections present at admission to the department; (%), count

During the study period, 63 UTIs were noted at admission. They were more frequently observed in females 42/956 (4.21%) than in males 21/1732 (1.2%), *p* = 0.0000004, and in internal 42/860 (4.88%) than in surgical 21/1891 (1.11%), *p* < 0.0000001. No statistically significant difference was observed in terms of the mean LOS at ICU of patients with UTI during admission (14.33 days CI 95% [10.71–17.95]) and the rest of the observed individuals (12.99 days CI 95% [12.28–13.69]) *p* = 0.468. Mortality was higher in patients with UTI present during admission (24/63; 38.10%) compared to both the overall patient population without UTI at admission (842/2688; 31.32%; *p*= 0.239) and patients with ICU-acquired CAUTI (56/207; 27.05%; *p*= 0.093). However, these differences were not statistically significant. Microbiological analysis identified 62 findings of etiological factors. Among these, the most common was *Escherichia coli* 16/62 (25.8%). Although alert pathogens were a common finding, 21/62 (33.87%), they were less frequently found in comparison to CAUTI pathogens 134/259 (51.74%), (63.68%), *p* = 0.01. Gram-negative strains with ESBL resistance mechanism constituted for 7/62 (11.29%) and with MBL for 4/62 (6.45%) Observed etiological factors are depicted in Fig. 7.

### CAUTI preventative measures realization assessment

This study evaluated the adherence to preventive measures for CAUTI. [20, 21] 1176 preventive care protocols were collected. The overall mean compliance with the assessed preventive elements was 88.36% CI 95% (86.99–89.72%). The least adhered-to measures included avoiding pressure on the urinary catheter by the patient’s body, with compliance of 83.26%, and providing maximal barrier precautions during the insertion achieved in 83.74% of observed cases. The highest compliance rate of 99.13% was observed for the use of a sterile closed urinary catheter system and placement of the urine collection bag below the bladder at 94.78%. The strongest negative correlation between adherence to the bundle protocol criteria and CAUTI density in corresponding months was -0.32, which was observed for applying single-use lubricant; however, neither this correlation (*p* = 0.157) nor any other corresponding relationship proved statistically significant (*p* = 0.157–0.967). Findings of the preventative packages monitoring are listed in Table 6.

**Table 6:** Findings of control of CAUTI preventative bundles sorted from the most to the least frequently fulfilled criteria [20, 21]

CAUTI bundle prevention criterium	% of fulfilled criteria	CI 95%
Sterile closed drainage system	99.13	98.41–99.85
Urinary collecting bag below the level of the bladder, hanging beside the bed	94.78	92.20–97.36
Urinary collecting bag with less than 75% of capacity full	89.13	83.58–94.68
Presence of securement of the catheter	87.96	82.69–93.22
Urinary catheter never disconnected	83.87	80.66–87.08
Single-use lubricant used	83.83	80.05–87.60
Maximal barriers precautions when inserted	83.74	79.98–87.50
Avoiding pressure on catheter - urinary catheter above the leg	83.26	79.81–86.72

## Discussion

Our study aimed to comprehensively evaluate CAUTIs in the ICU setting, focusing on multiple critical aspects, including incidence rates, infection characteristics, and microbiological profiles. The investigation also encompassed an analysis of antibiotic resistance patterns and an assessment of preventative measure compliance. This multifaceted approach was designed to provide a thorough understanding of CAUTI dynamics in critical care environments and identify potential areas for improvement in infection prevention strategies. Contextualization of findings within the broader perspective of international literature and previous research enables a better understanding of observations.

The first issue analyzed in this research was the assessment of CAUTI frequency based on epidemiological indicators. Globally, the incidence of CAUTIs varies, influenced by factors such as healthcare infrastructure, infection control practices, and regional microbial flora.

ECDC’s Annual Epidemiological Report (AER) of HAI in European ICUs for 2020 found a mean UTI incidence rate of 3.0 (median: 1.4, IQR: 0.0–4.4), and 95.2% of these infections were associated with urinary catheters. The mean incidence density of CAUTIs in ICU patients was 4.0 episodes per 1,000 Uct-D (IQR: 0.0–6.0) [22]. Both of these epidemiological indicators are also around twice as low as the CAUTI rate and density found in our department in 2020, respectively 7.31 (median: 6.8, IQR: 4.61–9.01) and 7.4 (median: 6.82, IQR: 4.69–9.13) The 2021 ECDC AER showed a mean UTI incidence rate of 3.6 episodes per 1,000 Pt-D (median: 2.3, IQR: 0.6–5.6), of which 97.3% were associated with the presence of urinary catheters. The device-adjusted incidence density for CAUTI in ICU patients was 4.4 episodes per 1,000 Uct-D (IQR: 0.7–6.7) [6]. Those indicators for 2021 in our study equaled 8.59 (median: 6.82, IQR: 6.59–10.52) and 8.66 (median: 8.95, IQR: 6.61–10.58) respectively. The increasing trends from 2020 to 2021 appeared in both ECDC reports and our unit. Nevertheless, ECDC’s findings are still almost twice less than in this research. Italy, out of the AERs’ countries, exhibited the highest mean incidence rates of CAUTI in both years, respectively 5.1 (Median: 2.4, IQR 0.0–4.0) and 4.7 (median 2.8, IQR 0.00–5.4) [6, 22]. From 2015 to 2019, the USA CDC’s NHSN, the primary HAI surveillance system [23], recorded consistent and significant decreases in CAUTI incidence, then, due to the pandemic, a significant increase was observed in 2020 compared to 2019, with a 19% rise during the fourth quarter [24]. After the pandemic, the CAUTI trends were observed to return to the declining trend. The CDC HAI progress report indicated CAUTI rates to drop by 16% across the USA’s ICUs between 2022 and 2023 [25].

The mean CAUTI incidence density for years 2020 - 2023 across all states’ ICUs interfered from the NHSN CDC-Acute Care Hospital HAI Progress Reports equals 0.91 CI 95% (0.87–0.95), which is almost eight times lower than 7.22 CI 95% (6.27–8.17) in our unit in the same time, *p* < 0.000001 [25–29].

International Nosocomial Infection Control Consortium (INICC) HAI report for 45 countries from Asia, Africa, the Middle East, Eastern Europe, and Latin America in 2015 - 2020 for adult and pediatric ICU patients found a CAUTI density of 2.91 [30]. Another INICC study for 37 countries of the aforementioned geographical regions in 2014 - 2022 established a pooled CAUTI density of 3.93; 14.03 in Eastern Europe and 6.28 in Asia [31].

The findings of this research were further compared to the results of studies performed in the same department in the previous years. Between 2007 - 2010 CAUTI mean incidence density was 4.8 (95%CI: 3.5–6.5) [32]. Throughout 2012 - 2014, it equaled 6.81 (IQR: 3.02–9.18), while in 2015 - 2017 it was 6.5 ± 1.2 [33, 34]. Having taken into consideration the fact that we established the mean CAUTI density of 7.04 ± 3.18 (95% CI: 6.18–7.91; IQR 5–9) for the total studied period, a disturbing trend becomes apparent.

Certain studies demonstrate CAUTI incidences exceeding those observed in our investigation. In a single-center, retrospective cohort study conducted at another ICU of an academic hospital in Poland between March 2020 and July 2021, CAUTI density was 15.8, around twice higher than the mean incidence density of 7.6 CI 95% (5.45 - 9.76) extracted from our data to match the same period [35]. CAUTIs show significantly higher rates across African healthcare settings compared to our and global averages, with prevalence rates ranging from 15.7 to 28.1 per 1000 catheter days and a pooled prevalence of 43.28%, predominantly caused by multidrug-resistant *Escherichia coli* (34.6–45.06%), Klebsiella spp., and *Pseudomonas aeruginosa* [36–41].

The findings of significantly higher CAUTI frequency in females and medical patients correspond to the findings of other similar studies [31, 35, 42, 43]. Among the risk factors that were not assessed in this study, increased CAUTI risk was proved to be associated with states such as paraplegia, Eastern European setting, suprapubic catheters, elder age, pre-infection LOS, higher urinary catheter-device utilization ratios, public facilities, and neurologic ICU settings [31, 44]. These findings underscore the need for strategies to reduce LOS, urinary catheter utilization, and targeted CAUTI prevention in high-risk populations and settings.

The next issue analyzed in this study was the analysis of LOS. Several findings regarding the impact of CAUTI on hospitalization duration were revealed. Notably, there were no statistically significant differences in LOS between the pandemic and post-pandemic periods, suggesting that the COVID- 19 pandemic did not substantially affect the duration of ICU stays in our center. A striking observation was made when comparing the LOS between patients with and without CAUTI. Patients who developed CAUTI had a substantially longer hospitalization, with high statistical significance. This may indicate that CAUTI extends the period of hospitalization but can also be the result of the CAUTI risk increasing along with prolonged catheterization in patients hospitalized longer. Arguments for both theses can be found in the literature. Research indicates that CAUTIs are linked to longer hospital stays for patients [42]. Specifically, it was found to extend the length of ICU stay by an average of 1.59 days (CI 95% 0.58–2.59 days) [45]. However, our observed difference in LOS was notably larger. On the other hand, prolonged catheterization is the most significant risk factor for CAUTI, and the risk rises non-linearly with each extra day a catheter remains in place [44, 46]. The substantially longer LOS observed in our study compared to previous reports might be attributed to the complexity of cases treated in our center or other local factors requiring further investigation.

Our mortality analysis revealed several important insights. The comparable mortality rates between patients with and without CAUTI suggest that in our ICU population, it may not be a primary driver of mortality. This finding held across various subgroup analyses, including gender and clinical context (surgical vs medical). Notably, even the presence of drug-resistant organisms in CAUTI cases did not significantly impact mortality rates, possibly reflecting appropriate antibiotic choices and proper interventions in our ICU. These findings contrast with some previous studies. Literature indicates that mortality increases in patients with CAUTI [42]. Furthermore, this infection was found to increase the risk of death by 15% (CI 95%: 3–28%) (although authors claim that this may be accidental) [45] and is described as responsible for approximately 20% of healthcare-acquired bacteremia episodes in acute care facilities [47]. A 24-year multinational, prospective cohort study at 786 ICUs aiming to identify mortality risk factors in ICU patients identified CAUTI as a mortality risk factor with an adjusted odds ratio of 1:18 [48]. The divergence in our findings might be attributed to our center’s strict adherence to catheter management protocols and appropriate antimicrobial stewardship.

The subsequent phase of our research examined the CAUTIs in the context of the COVID- 19 pandemic. Since ECDC has not yet published appropriate data for years later than 2021, our post-pandemic CAUTI epidemiological indicators were compared to ECDC’s pre-pandemic 2019 AER. The urinary catheters were implicated in 94.0% of UTI episodes, mean incidence rate of UTIs was 2.2 episodes per 1,000 Pt-D (IQR: 0.0–3.0), while the mean density was 2.8 episodes per 1,000 Uct-D (IQR: 0.0–4.0) [49]. The pandemic CAUTI epidemiological indicators, therefore, turned out to be higher both in ECDC reports and in our study since after the pandemic we found a mean CAUTI rate of 6.22 (median 5.82, IQR: 4.54–7.83) and a mean incidence density of 6.25 (median 5.9, IQR 4.55 - 7.88). Nevertheless, the differences we observed were of marginal statistical significance and should be interpreted with caution. Furthermore, the similar CAUTI rates between COVID-positive and COVID-negative patients (10.23% vs 9.18%, *p*= 0.942) indicate that SARS-CoV- 2 infection itself may not predispose patients to increased risk of CAUTI.

Elevated rates of HAIs in American ICUs were observed, including a 30% increase in CAUTI incidence during the pandemic [10]. NHSN CDC HAI progress reports show significantly greater mean CAUTI density during the pandemic 1.05 CI 95% (0.99–1.11) in comparison to the post-pandemic 0.76 CI 95% (0.72–0.81), *p* < 0.0000001 [25–29].

Interestingly, a reverse relationship was observed in Saudi Arabia. A retrospective analysis of data spanning 2019 - 2021 and covering adult ICUs in 78 hospitals revealed a significant reduction in CAUTI rates during the pandemic (2020 - 2021) compared to 2019, with densities dropping from 1.54 to 0.96 per 1000 Uct-D [50].

Other studies found no statistical significance in CAUTI incidence density differences prior to and during the pandemic [51–54].

The paradoxical or seeming decrease in CAUTI rates in these studies could be attributed to reduced surgical and elective admissions during the pandemic, which could have reduced the number of patients susceptible to CAUTI. Moreover, the increased antibiotic use for COVID- 19 patients could mask bacteriuria, and a potential reduction in performed urine cultures minimizing personnel exposure to COVID- 19 patients could underestimate that rate [50].

A further point of interest in this study was the analysis of CAUTI etiological factors. According to the ECDC AERs throughout years 2019, 2020 and 2021 the microbiological profile of the organisms isolated from ICU patients with UTIs remained relatively uniform - the three most frequently found pathogens were *Escherichia coli* (27.1–31.1%), *Enterococcus* spp. (20.5–23.6%) and *Pseudomonas aeruginosa* (14.3–14.6%) [6, 22, 49]. The top 3 most frequent CAUTI pathogens in the USA ICUs Reported to NHSN in 2018 - 2021 were *Escherichia coli* (33.5%), Klebsiella spp. (14.5%) and *Pseudomonas aeruginosa* (13.4%) [55].

At our department, in the years 2012 - 2014, the most frequent CAUTI pathogens were gram-positive bacteria (23%), *Acinetobacter baumannii* (20%), and *Klebsiella pneumoniae* (18%) [33]. Then, in 2015 - 2017, the most common CAUTI etiological factors were *Acinetobacter baumannii* (31%), *Enterococcus* spp. (20%), *Klebsiella pneumoniae* and *Escherichia coli* (both 14%) [34].

Differences in the microbiological profile of CAUTI can be spotted between separate units from the same country in the same period. In a single-center, retrospective cohort study conducted at another university hospital’s ICU in Poland between March 2020 and July 2021, CAUTIs’ most common pathogens were *Klebsiella pneumoniae* (36.4%), *Enterococcus faecalis* (22.1%), and *Enterococcus faecium* (14.3%), which stands out from our findings [35]. Nevertheless, a common feature for that and our departments’ CAUTIs is the predominance of multidrug-resistant organisms - respectively 68.8%. and 62.07% [35].

The World Health Organization (WHO) Antimicrobial Resistance (AMR) Surveillance survey assessing the effects of the COVID- 19 pandemic on AMR surveillance, prevention, and control revealed that around 40% of observed countries reported increases in MDR HAIs and MDR infections due to the COVID- 19 pandemic [9].

A CDC report revealed a substantial increase in AMR in USA hospitals during the COVID- 19 pandemic, with at least a 15% rise in resistant infections from 2019 to 2020, including sharp increases in carbapenem-resistant *Acinetobacter* (78%), carbapenem-resistant Enterobacteriaceae (35%), and ESBL-producing Enterobacteriaceae (32%) [56].

According to the ECDC’s AERs, the percentage of alert pathogens among HAI etiological factors in the ICUs rose from 18.82% in 2019 to 23,35% during the pandemic (2020 - 2021) [6, 22, 49]. None of these registers assessed the increase in resistance separately for CAUTI; therefore, we cannot compare these studies with our results.

In this analysis of CAUTI in the ICU setting, we observed notable differences in AMR patterns between the pandemic and post-pandemic periods. The markedly higher percentage of alert pathogens during the pandemic aligns with global concerns about antimicrobial resistance in this period. This observation may reflect the intensive antibiotic use during the pandemic, particularly in critically ill COVID- 19 patients.

Further analysis revealed that CAUTI caused by alert pathogens occurred approximately 9 days later. This delayed emergence of more resistant strains might have occurred because the initial antibiotic treatments potentially administered before created selective pressure, allowing resistant subpopulations to survive and proliferate while eliminating more susceptible bacteria.

The analysis of UTIs present upon admission to the department indicated a high percentage of drug-resistant etiology. The potential source of those organisms might have likely been the flora of other departments of the hospital, as several patients with UTI upon admission had undergone urological procedures. That proves the necessity to undertake further measures against the development of multidrug-resistant organisms also outside of the ICU setting.

In 2015, the NHSN revised its CAUTI definition to include only infections with identified bacterial causative agents. This change excludes *Candida* spp. as a standalone CAUTI etiology [57]. Studies examining the impact of this change showed a 25 - 50% reduction in CAUTI density [58–60]. Despite that, authors still recommend listing *Candida* spp. findings from urine culture [59]. We included *Candida* strains among other pathogens because their co-finding might have proven valuable in depicting the complete microbiological landscape of CAUTI. This methodological difference could have contributed to our higher rates when compared with US data that follows the 2015 NHSN definition. However, when comparing with European surveillance data from ECDC the differences in reported rates cannot be attributed to this definitional variation, since ECDC HAI-Net ICU protocol does not mention *Candida* spp. exclusion from CAUTI diagnosis criteria and *Candida* spp. is frequently displayed among pathogens isolated in UTI episodes among ICU patients [15, 22, 49].

The last issue analyzed in this study was CAUTI prevention. To address the significantly higher rates of HAIs, the INICC developed Multidimensional Approach (IMA) consisting of intervention bundles, education/training, surveillance, process monitoring, performance feedback, and regular rate reporting [61]. This strategy has demonstrated success in reducing CAUTI by 37% in adult ICUs [62]. The approach’s effectiveness derives from combining multiple prevention strategies rather than single measures [7]. Care “bundles” consist of straightforward collections of evidence-based practices that, when applied together, enhance the consistency of their execution and lead to better patient outcomes [63].

The CAUTI rates may be successfully decreased without significant additional resources or increased medical personnel. The IMA implementation achieved significant CAUTI reductions: 89% across 299 ICUs in 32 low-income or middle-income countries (LMICs) [20] and 57% in 10 pediatric ICUs across 6 developing countries [64] with similar outcomes reported in additional studies [65–67]. These findings demonstrate this cost-effective approach’s feasibility and potential scalability for CAUTI prevention in ICU settings.

The adherence to the bundle protocols observed in our study did not differ from the compliance to the prophylactic measures observed through 3 months in 2014 in the same unit [33]. The high incidence of CAUTI in our study, despite high compliance with preventive elements along with no correlation found between both findings, may result from the fact that certain preventive measures, such as catheter site care, assessment of catheter disconnection, and urine backflow from tubes to the bladder in patients requiring transport to the operating room or diagnostic laboratory, should be monitored continuously rather than twice a week for proper evaluation. The maintenance of urinary catheters in patients with anuria where the presence of the catheter was not necessary also has a significant impact on UTI occurrence.

The INICC recommends that urinary catheters should only be inserted when essential for patient care and removed as soon as the clinical indications no longer persist [63]. ICU care alone is not a sufficient reason for urinary catheter placement; a clear clinical indication is necessary. Alternative bladder management techniques, like intermittent catheterization should be considered, when appropriate [68]. Regular catheter changes as a preventive measure against CAUTI are not advised [63]. More evidence and studies are needed to establish whether the routine replacement of indwelling urinary catheters that have been in place for over 30 days should be recommended practice as an infection prevention measure [68].

Research studies highlight the urgent need to strengthen educational initiatives in LMICs [40]. Regular in-service education, training programs, and guaranteed access to essential materials and supplies are crucial for improving healthcare outcomes and infection prevention. Enhancing the knowledge and practices of nurses is essential for improving patient outcomes [41].

The study has several notable limitations. First, being a single-center study, the findings may not be generalizable to other healthcare settings. Second, the microbiological profile specific to our ICU differs from other institutions, potentially affecting the applicability of our results. Third, mortality outcomes in ICU patients are influenced by multiple confounding factors, making it challenging to isolate the direct impact of CAUTI. Fourth, following our methodology, we did not analyze the occurrence of sepsis secondary to urinary tract infections, which could have provided additional insights into severe complications.

Furthermore, the study did not include detailed patient characteristics such as age, specific comorbidities, and particular reasons for ICU admission. This approach was chosen as we primarily aimed to evaluate temporal changes in CAUTI epidemiology between pandemic and post-pandemic periods rather than conduct a comprehensive risk factor analysis. Our surveillance-based methodology followed standard ECDC protocols focusing on infection rates, pathogens, and resistance patterns. Given our large sample size of 2,751 patients, comprehensive collection of detailed clinical data would have exceeded available resources, particularly during pandemic conditions when research capacity was constrained. Instead, we prioritized assessment of potentially modifiable infection control practices over non-modifiable patient characteristics, an approach that better aligns with actionable infection prevention strategies.

Additionally, our CAUTI prevention protocol adherence assessment was conducted only at specific time points, with the number of observations being disproportionate to the total number of catheterized patients. Protocol criteria fulfillment was verified only at the moment of observation, and continuous monitoring would have not been feasible. This point-in-time assessment means that elements such as urinary bag positioning below the bladder level or catheter disconnection status might have changed between observation periods, potentially underestimating or overestimating actual protocol adherence rates.

Finally, in COVID- 19 patients, due to the safety measures we could not evaluate adherence to CAUTI prevention bundles, which might have affected the observations.

Throughout the study period, a consistent gender disparity was observed in ICU admissions, with males comprising the majority of the total patient population. This disproportion reflects the epidemiological characteristics of critically ill patients requiring intensive care at our institution during the study timeframe. Patient admission was based exclusively on clinical indications and medical necessity, without any gender-based criteria influencing admission decisions. While various surgical subspecialties including urology were represented in our cohort, specialty-specific admissions did not substantially contribute to the overall gender distribution observed in this study.

## Summary

This prospective single-center study revealed that CAUTI acquired in the ICU had a high incidence rate, affecting approximately 9% of ICU patients. The comparison of pandemic versus post-pandemic incidence densities demonstrated marginally significant statistical differences. CAUTI occurred more frequently in male and medical patients but did not influence mortality rates. Patients who developed CAUTI exhibited significantly longer LOS. Alert pathogens comprised the majority of etiological agents and were isolated more frequently during the pandemic compared to the post-pandemic period; however, this had no impact on mortality. Systematic CAUTI surveillance, implementation of preventive measures, and ongoing evaluation are essential for improving the hospital’s epidemiological status, patient safety, and clinical outcomes related to CAUTI.

## Data Availability

The individual participant data that underlie the results reported in this article, after de-identification, are not openly available due to the sensitive nature of clinical data containing protected health information from intensive care unit patients. However, these data are available from the corresponding author upon reasonable request. Researchers seeking to access the data must provide a methodologically sound proposal to achieve the objectives outlined in the approved proposal. Data access will require signing a formal data access agreement. The data are stored in controlled access data storage at Wroclaw Medical University. All studyrelated documents, including relevant text, tables, figures, and appendices, will be made available alongside the dataset. The data will be accessible immediately following article publication and remain available for 5 years thereafter.

## Abbreviations

HAIs: Healthcare-associated infections
UTIs: Urinary tract infections
CAUTI: Catheter-associated urinary tract infection
ICUs: Intensive care units
LOS: Length of stay
Uct-UR: Urinary catheter utilization ratio
ECDC: European Centre for Disease Prevention and Control
CDC: Centers for Disease Control and Prevention
NHSN: National Healthcare Safety Network
MDR: Multidrug-resistant
XDR: Extensively drug-resistant
PDR: Pandrug-resistant
ESBL: Extended-spectrum beta-lactamase
MBL: Metallo-beta-lactamase
VIM: Verona integron-encoded metallo-beta-lactamase
NDM: New Delhi metallo-beta-lactamase
OXA- 48: Oxacillinase-48
GRE: Glycopeptide-resistant enterococci
VRE: Vancomycin-resistant enterococci
MRSA: Methicillin-resistant Staphylococcus aureus
VISA/VRSA: Vancomycin-intermediate and vancomycin-resistant Staphylococcus aureus
AER: Annual Epidemiological Report
WHO: World Health Organization
AMR: Antimicrobial Resistance
IMA: INICC Multidimensional Approach
INICC: International Nosocomial Infection Control Consortium
LMICs: Low-income or middle-income countries
